# Transumbilical laparoscopy for pneumoperitoneum establishment: a comprehensive multicentre evaluation affirming safety, feasibility, and a range of clinical benefits

**DOI:** 10.3389/fsurg.2024.1390038

**Published:** 2024-04-22

**Authors:** Antonia Rizzuto, Cristina Bozzarello, Jacopo Andreuccetti, Angela Amaddeo, Antonio Maria Iannello, Carlo Sagnelli, Roberto Cirocchi, Diego Cuccurullo, Giusto Pignata, Francesco Corcione

**Affiliations:** ^1^ Department of Medical and Surgical Sciences, Magna Græcia University, Catanzaro, Italy; ^2^ Department of General Surgery, Civil Hospital of Brescia, Brescia, Italy; ^3^ Department of General, Mininvasive and Robotic Surgery, Colli Monaldi Hospital, Naples, Italy; ^4^ Department of Medicine and Surgery, University of Perugia, Perugia, Italy; ^5^ Department of General Surgery, Federico II University Hospital, Naples, Italy

**Keywords:** transumbilical laparoscopy (TUL), periumbilical laparoscopy, laparoscopic cholecystectomy, incisional hernia, pneumoperitoneum insufflation

## Abstract

**Introduction:**

Transumbilical laparoscopy (TUL) has emerged as a promising technique for establishing pneumoperitoneum in laparoscopic cholecystectomy, offering potential safety, feasibility, and clinical benefits. This retrospective multicentre study aims to evaluate the efficacy and outcomes of TUL in the management of gallbladder diseases.

**Methods:**

A retrospective analysis was conducted on a cohort of 2,543 patients who underwent TUL between 2011 and 2021 across various medical institutions in Italy. Data collection included demographic, clinical, intraoperative, and postoperative parameters. Standardized protocols were followed for preoperative and postoperative management. The TUL technique involved precise anatomical incision and trocar placement.

**Results:**

The study demonstrated favorable outcomes associated with TUL, including a low conversion rate to open surgery (0.55%), minimal intraoperative complications (0.16%), and short hospital stays (average 2.4 days). The incidence of incisional hernias was notably low (0.4%). Comparison with existing literature revealed consistent findings and provided unique insights into the advantages of TUL.

**Discussion:**

Despite limitations, such as the absence of a control group and the retrospective nature of the study, the findings contribute valuable insights to the literature. They inform surgical decision-making and advance patient care in laparoscopic cholecystectomy for gallbladder diseases.

**Conclusion:**

Transumbilical laparoscopy shows promise as a safe and feasible technique for establishing pneumoperitoneum in laparoscopic cholecystectomy. The study's findings support its clinical benefits, including low conversion rates, minimal complications, and short hospital stays. Further research, including prospective studies with control groups, is warranted to validate these results and optimize patient outcomes.

## Introduction

Minimally invasive surgery represents a cornerstone of 20th-century medical progress. Over the past two decades, it has predominantly replaced the open approach, offering a multitude of recognized advantages such as decreased postoperative pain, expedited recovery, and shorter hospitalization periods (1).

Laparoscopy hinges on the establishment of pneumoperitoneum, accomplished by infusing the peritoneal cavity with carbon dioxide (CO2) at a flow rate of 4–6 L/min until a pressure of 10–20 mmHg is attained (2). This pneumoperitoneum effectively converts a theoretical space into a palpable one, enabling meticulous anatomical dissection of tissues, thereby improving visibility and maneuverability at the surgical site (3).

According to several authors, the initiation of pneumoperitoneum is frequently considered the most challenging phase of laparoscopy. This step carries numerous potential complications, irrespective of whether the closed or open technique is employed, which may include vascular and visceral injuries (4).

The selection of the pneumoperitoneum creation technique can impact not only the incidence of intraoperative complications but also the severity of postoperative comorbidities, such as incisional hernia (5). Over the years, a variety of entry techniques have been developed to prevent and mitigate entry complications. Currently, the most used entry methods are the closed and open techniques (6).

The closed technique employs the Veress Needle, fitted with an inner obturator, inserted at Palmer's Point, situated about 2 cm below the left costal margin along the midclavicular line. Following insufflation of CO2, trocars are then introduced (7, 8).

The alternative approach entails using the Hasson trocar, a blunt cannula inserted into the peritoneal cavity through an incision, which can be positioned either periumbilically (PUI) or transumbilically (TUL) (8–10).

In the transumbilical approach, surgeons typically create a vertical or transverse incision that spans the entire length of the umbilicus. Conversely, the periumbilical technique involves making a transverse or *U*-shaped incision either above or below the umbilicus. Both methods have their proponents. Transumbilical incisions may offer benefits because the abdominal wall layers converge at the umbilicus, allowing for easier and quicker placement of laparoscopes and closure of the incision. Moreover, from an aesthetic standpoint, scars resulting from transumbilical incisions are less noticeable and concealed within the umbilicus.

However, transumbilical incisions pose a risk of increased surgical site infections due to the umbilicus's susceptibility to bacterial colonization (4, 11).

Despite the wealth of literature on the subject, a consensus has yet to be reached regarding the standard approach for abdominal access. Western surgeons tend to favour the periumbilical approach, while their Eastern counterparts prefer transumbilical approach (12). In the current paper we report our multicentric experience of TUL entry technique, in the last 10 years. Over the past 15 years, our practice has solely relied on the transumbilical approach for abdominal access during laparoscopic cholecystectomy. This observational study includes all patients who underwent transumbilical laparoscopy (TUL) during the last decade. Because of our steadfast utilization of the transumbilical approach, a control group employing the periumbilical approach is unavailable. Nevertheless, this study represents the most extensive case series in the literature regarding the use of the transumbilical approach for pneumoperitoneum induction. The purpose of this study is to describe the steps of the technique that we have standardized over the years and to assess its appropriateness and feasibility in terms of intraoperative and postoperative outcomes, including hospital stay, incisional hernia, and wound infection, in a group of patients who underwent laparoscopic cholecystectomy.

## Material and methods

### Study design

A retrospective study was conducted involving 2,543 patients who underwent surgery for gallbladder diseases (acute and chronic cholecystitis, gallbladder stones) between 2011 and 2021. Data collection and analysis were carried out at Ospedale Monaldi A.O.R.N dei Colli, Naples, Italy; University Federico II, Naples, Italy; and University Magna Graecia of Catanzaro, Italy (Table 1).

**Table 1:** Demographic data of patients undergoing surgery for gallstones disease through TUL.

Demographic data of patients undergoing surgery for gallstones disease	
Age (year)	Mean: 54 years
	(18–90 years)
Sex (%)	M: 37%
	F: 63%
Body Mass Index	Mean: 31
	(20–42)
Previous abdominal surgery (%)	18%
Acute cholecystitis (%)	20%
Chronic gallbladder diseases (%)	80%

### Participants and location

The study included adult patients aged 18 and above without contraindications for elective laparoscopic surgery. Demographic and clinical data were obtained from CPT codes at the mentioned medical institutions in Italy.

### Data collection

A comprehensive set of data was collected, including demographic information, clinical parameters, preoperative diagnoses, operative details (operative time, procedure performed, conversion to open surgery, intraoperative complications), and postoperative outcomes [complications according to Dindo-Clavien (13) classification, postoperative ileus, hospital stay, reintervention, biliary leakage, mortality]. Routine preoperative examinations and specific informed consent for surgery were obtained for each patient. The study adhered to the STROCSS Guideline 2021 (14).

### Preoperative and postoperative management

No dietary restrictions were imposed before surgery. Prophylaxis against deep venous thrombosis (DVT) was achieved through early mobilization, compression stockings, and administration of Low Molecular Weight Heparin (LMWH). Patients were encouraged to ambulate one hour post-surgery, with compression stockings removed six hours later. Administration of LMWH was tailored according to patients' body surface area (BSA) and continued for up to 15 days post-surgery. Antimicrobial prophylaxis with Cefazolin 1 gram was administered intravenously one hour before surgical incision. In cases of cefazolin allergy, alternative antibiotic options such as Clindamycin and Vancomycin were selected based on the severity of the allergy and specific patient factors. Dietary intake and discharge criteria were standardized, with patients typically discharged on the first or second postoperative day based on specific criteria. Our standard follow-up protocol for assessing the occurrence of incisional hernia consisted of a series of appointments scheduled at predetermined intervals: 1–2 weeks, 4–6 weeks, 3 months, 6 months, and then annually. Furthermore, we provided patients with education regarding the signs and symptoms of incisional hernia, urging them to promptly report any new concerns or symptoms. This thorough follow-up approach enabled us to identify any potential issues at an early stage and take necessary interventions to enhance patient outcomes.

### Laparoscopic surgical technique

**Figure 1:**
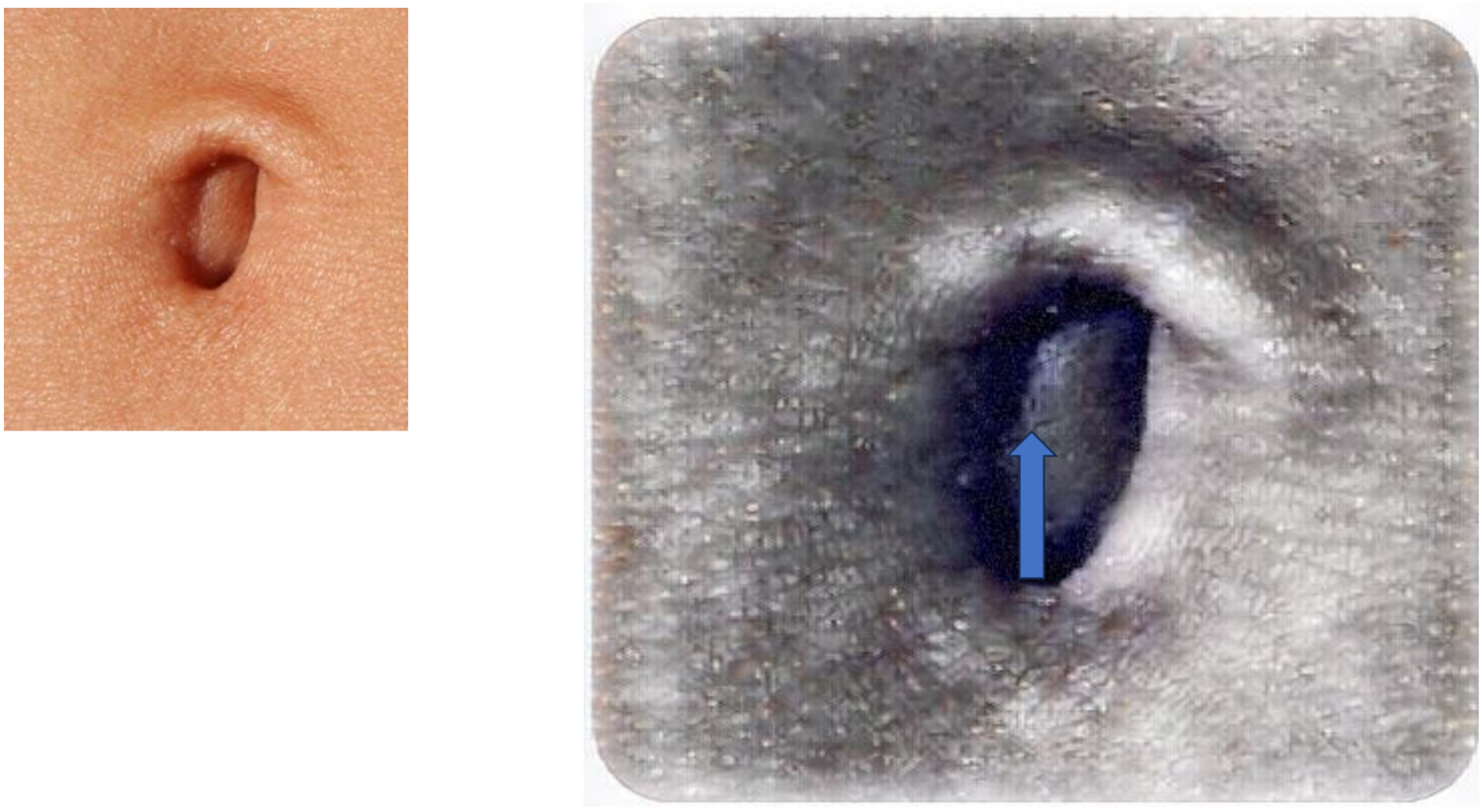
Identification of the deep point of the navel by gripping the upperside of the umbilical ring.

**Figure 2:**
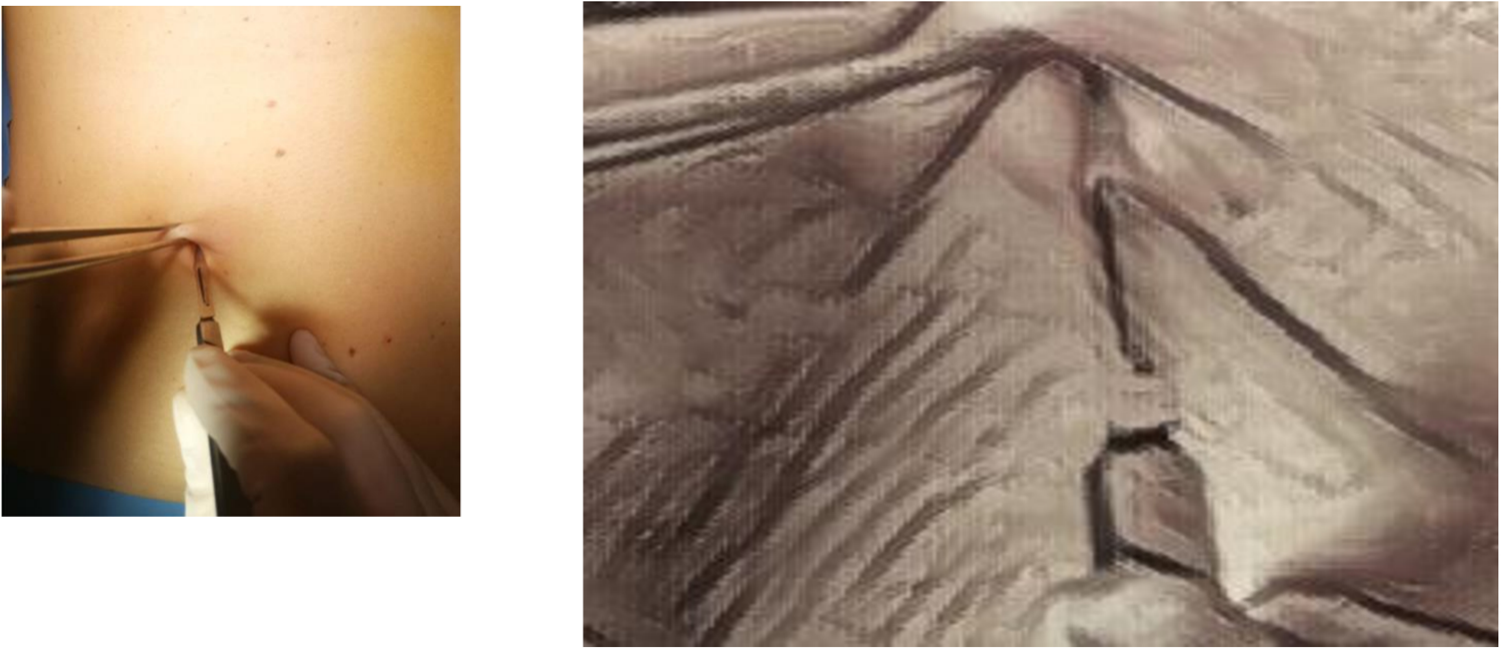
Longitudinal incision.

The surgical technique to perform TUL (trans umbilical laparoscopy) has been standardized as described in Table 2. (Supplementary Video S1). After the identification of the navel (Figure 1) a longitudinal or semilunar incision is meticulously executed, aligning precisely with the anatomical location of the umbilical scar. (Figure 2) At this juncture, we encounter the intersection of the aponeurotic fascia and the parietal peritoneum, where these two structural layers come into intimate contact (15). To achieve a precise dissection and maintain surgical precision, a surgical forceps is employed to gently elevate the umbilicus, facilitating the precise delineation and identification of the umbilical scar before commencing the incision, a visual representation of the procedure can be observed in *Image 1*. This meticulous approach ensures that the incision is not only accurately positioned but also conducted with utmost precision. Besides, the longitudinal incision, which plays a pivotal role in this surgical procedure, is precisely calibrated to measure approximately 15 ml. This carefully calculated incision size ensures that the surgical access point is optimal, allowing for the successful execution of the procedure while maximizing the cosmetic outcome. A notable aspect of this procedure is the minimal dissection required due to the absence of subcutaneous fat in the area. This unique anatomical characteristic simplifies the surgical process, allowing for a more direct approach to the underlying structures. Subsequently, the surgical protocol proceeds as follows: the parietal peritoneum is meticulously incised using a cold cutter and a disposable 10- or 12-ml trocar is introduced trough the umbilicus.

**Table 2:** Surgical steps for the technical execution of trans-umbelical laparoscopy.

Surgical steps of TUL	
1	Indentification of the deep point of the navel by gripping the upper side of the umbilical ring.
2	Longitudinal (or semilunar, depending on the navel's surgery) intraumbilical incision of the skin.
3	Blunt dissection of the subcutaneous fat and tissues, preserving the vascularization of the navel and without interrupting the pedicle.
4	Visualization of the linea alba and blunt dissection of the trasversalis fascia and peritoneum, which are embryilogically fused at this point.
5	Inserting the surgeon's finger to confirm unrestricted entry into the intraperitoneal space.
6	Suture of the peritoneum and trasversalis fascia.
7	Aesthetic reconstruction of the subcutaneous and cutaneous layers.

Then, laparoscopic cholecystectomy (16, 17) was performed using the technique established by Corcione et al. (15). The following steps are, as always, the exposure of Calot's triangle establishing the critical view of safety (18); the isolation and clipping of cystic artery and duct; the gallbladder dissection.

**Figure 3:**
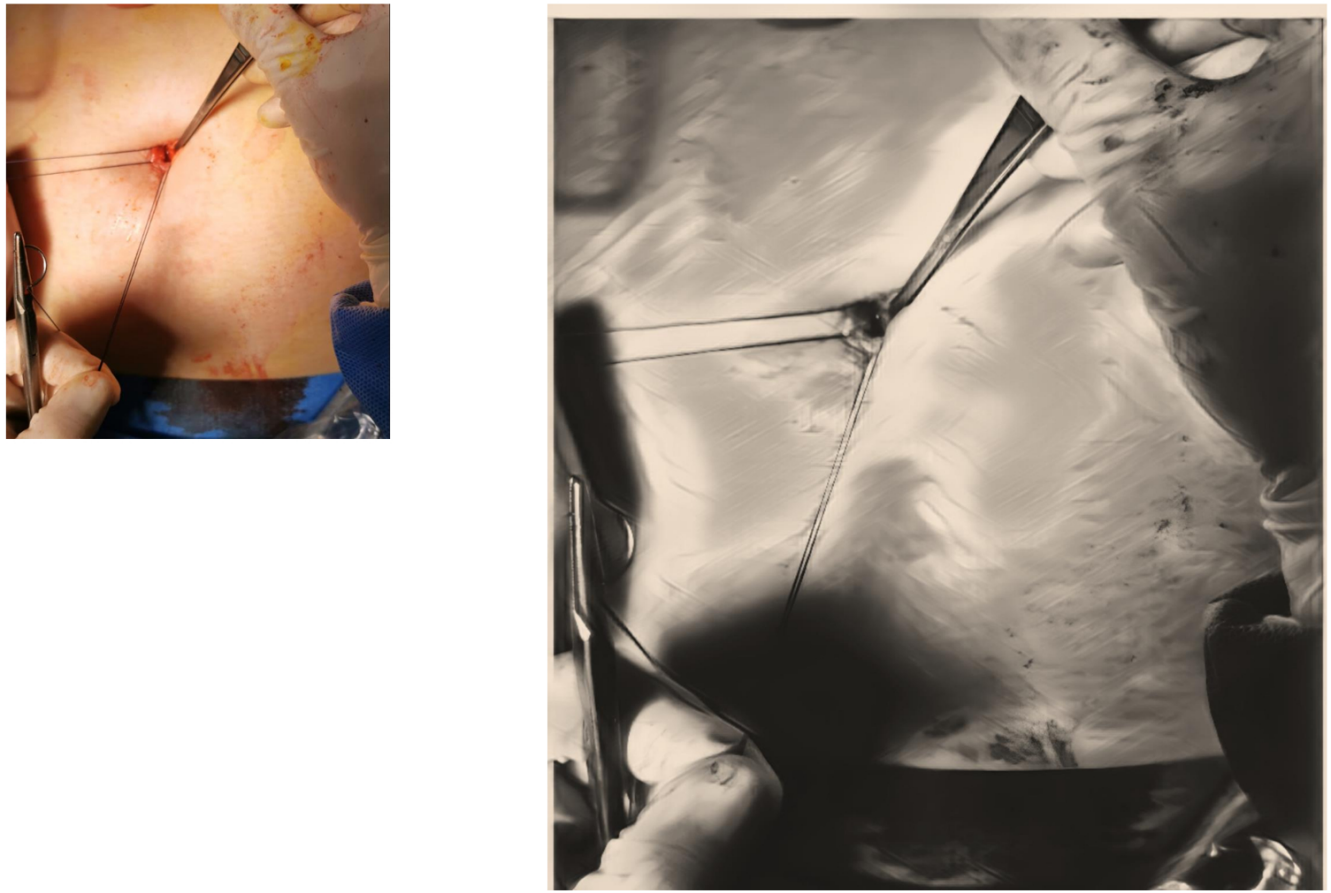
Suture of fascia and peritoneum in a single layer.

The extraction of the anatomical specimen consistently involves the use of an endobag in all cases. Following this, meticulous closure of the fascia surrounding the umbilicus is achieved using single layer suture with Vicryl 3.0, Figure 3 providing essential structural support. This suture, administered with a 5/8 needle, ensure precise reinforcement of the area. Subsequently, the edges of the skin around the umbilicus are meticulously approximated using absorbable sutures. Carefully placed to ensure even alignment, these sutures contribute to a symmetric and aesthetically pleasing appearance.

## Results

Between January 2011 and December 2021, 2,543 patients (963 males and 1,580 females) underwent laparoscopic surgery for gallstone disease. The study exclusively employed transumbilical laparoscopy access for entering the abdominal cavity in all recruited patients. Notably, no changes in the surgical technique for abdominal cavity entry were required. The majority of subjects exhibited symptoms of gallstone disease, primarily presenting with postprandial dyspepsia and/or biliary colic; some also had a history of acute biliary pancreatitis.

### Preoperative findings

A variegate group of 2,543 patients was analysed. Patients had an age between 18 and 90 years, with a median age of 54 years. The 37% of patients were males (933/2,543) and the 63% were females (1,610/2,543). The examined subjects had an average BMI of 31 kg/m^2^ (range: 20–42). All I-IV ASA score patients were included. In 18% (457/2,543) of patients pneumoperitoneum was induced by Verres assisted laparoscopy with a trans umbilical incision (TUL), since they had already undergone a median laparotomy.

20% of patients (508/2,543) had received a diagnosis of Acute cholecystitis while 80% (2,035/2,543) were diagnosed with Chronic Gallbladder Diseases. In the latter group we included both patients with chronic-recurrent cholecystitis and patients who were symptomatic for gallbladder stones with no evidence or history of previous cholecystitis (Table 1).

### Intraoperative findings

The average time for the surgery was 54 min, ranging from 20 to 270 min. Specifically, the average time it took to start the procedure was 2.3 min, ranging from 1.8 to 4 min. In 35% of cases (890 out of 2,543), intraoperative cholangiography was performed. Out of 2,543 patients, 14 (0.55%) required conversion to open surgery. See Table 3 for more details.

**Table 3:** Conversion to open surgery.

Conversion to open surgery	
Intaoperative diagnosis of Mirizzi Syndrome	2 patients
	(0.08%)
Uncontrollable bleeding from Hepatic Liver bed	4 patients
	(0.16%)
Complex surgical or non-surgical adhesions	7 patients
	(0.27%)
Peritoneal carcinomatosis for a concomitant ovarian cancer	1 patients
	(0.04%)
Total	14 patients
	(0.55%)

The average amount of blood lost during surgery was 75 ml, with a range from 10 ml to 380 ml. Among all the procedures, 4 patients (0.16%) experienced intraoperative complications. These complications included 3 cases of duodenal lesions and 1 case of splenic flexure lesion. There was one reported case (0.04%) of biliary duct injury. No vascular complications were observed (Table 4).

**Table 4:** Intraoperative data.

Intraoperative data of patients undergoing surgery for gallstones disease	
Operative time (min)	Mean: 54 min
	(20 min–270 min)
Entry time (min)	Mean: 2.3min
	(1.8 min–4 min)
Intraoperative cholangiography (%)	35%
Conversion to oper surgery (%)	0.55%
Blood loss (ml)	Mean: 75 ml
	(10 ml–380 ml)
Intraoperative complications (%)	0.16%
Biliary duct lesions (%)	0.04%
Vascular lesions (%)	0%

### Postoperative outcomes

The main postoperative discovery in this study was the relatively low occurrence of incisional hernias, with only 10 patients out of 2,543 experiencing this complication (0.4%). Additionally, 42 patients (1.65%) encountered port site infections following the procedure, although none developed seromas.

Postoperative ileus was documented within 0–3 days post-surgery. On average, patients stayed in the hospital for 2.4 days, with durations ranging from 1 to 72 days. Biliary leakage was observed in 5 patients (0.2%), all of whom received conservative treatment. The rate of reintervention was 0.36% (9 out of 2,543 patients), with 8 patients requiring further hemostasis and 1 patient suffering from an iatrogenic ileal injury within this subgroup. Mortality occurred in 0.08% of patients (2 out of 2,543), and it was not directly associated with surgical complications; one patient had severe renal impairment, and the other patient experienced multiple organ failure (MOF).

Complete data are summarized in Table 5.

**Table 5:** Postoperative data.

Postoperative data of patinets undergoing surgery for gallstones disease	
Dindo–Clavien	
Primary ileus (day)	0–3 days
Total hospital stay (day)	Mean: 2.4 days
	(1–72 days)
Biliary leakage (%)	0.2%
Mortality (%)	0.08%
Incisional hernia on TUI (%)	0.4%
Wound infection on TUI	1.65%
Seroma on TUI (%)	0%
Reinterventions (%)	0.36%

## Discussion

Our manuscript presents the most extensive case series of transumbilical laparoscopy (TUL) available in the medical literature to date. TUL stands as a pivotal surgical approach for abdominal entry, bearing significant importance. Surgeons presently rely on personal preferences to determine the method of peritoneal entry due to the absence of a definitive guideline (12, 19). However, in our clinical practice, we consistently employ the TUL technique to initiate pneumoperitoneum in procedures involving trocar insertion through the umbilicus, such as laparoscopic cholecystectomy.

Our study comprised a large cohort of 2,543 patients who underwent laparoscopic surgery for gallbladder diseases over a ten-year period. The inclusion criteria encompassed both acute and chronic cholecystitis, as well as gallbladder stones, ensuring a comprehensive representation of the patient population commonly encountered in clinical practice. This extensive sample size and broad inclusion criteria contribute to the robustness and generalizability of our findings.

Data collection in our study was meticulous, capturing demographic, clinical, preoperative, intraoperative, and postoperative parameters. This comprehensive approach enables a thorough analysis of various factors influencing surgical outcomes, enhancing the validity and reliability of our results. Our preoperative and postoperative management protocols were well-documented and followed standardized procedures, including prophylaxis against deep venous thrombosis, antimicrobial administration, and criteria-based discharge criteria. These standardized protocols ensure consistency in patient care and minimize variability in outcomes, strengthening the internal validity of our study.

The laparoscopic surgical technique employed in our study, specifically transumbilical laparoscopy (TUL), was meticulously described and standardized. The precise execution of the TUL technique, including anatomically precise incision and trocar placement, highlights attention to detail and surgical expertise. Moreover, the use of disposable trocars and adherence to established surgical protocols underscore the safety and reproducibility of the procedure, contributing to its applicability in clinical practice.

In terms of results, our study demonstrated favorable outcomes across various parameters, including surgical duration, intraoperative complications, conversion to open surgery, postoperative complications, and hospital stay. The low incidence of adverse events, such as incisional hernias and wound infections, reflects the effectiveness of the TUL approach and underscores its safety and feasibility in laparoscopic surgery for gallbladder diseases. This investigation builds upon prior research, including a meta-analysis conducted by Shih et al. (12), which compared various incision techniques for laparoscopic surgery. Noteworthy studies included in the meta-analysis encompass those by Bouffard-Cloutier et al. (20), Lee et al. (19, 21), Rafique et al. (22), Şentürk et al. (23), and Siribumrungwong et al. (1).

In our study, we observed a median age of 54 years among the analyzed patients, with a balanced distribution of gender (37% males and 63% females), reflecting the demographic diversity commonly encountered in gallstone disease populations. This finding aligns with previous studies by Bouffard-Cloutier et al. (20), Lee et al. (19), and Siribumrungwong et al. (1), which also reported similar age distributions and gender ratios among patients undergoing laparoscopic procedures for various abdominal conditions.

Regarding operative characteristics, our study demonstrated an average surgical time of 54 min, which is consistent with findings reported by Lee et al. who observed shorter surgical durations using the transumbilical approach compared to periumbilical incisions for cholecystectomy (34.2 ± 14.6 min vs. 41.7 ± 21.3 min, *P* = 0.020) (19). This result is also in accordance with a meta-analysis of 2020 (12).

In the meta-analysis by Shih et al. (12), which included data from three randomized controlled trials (RCTs), the transumbilical group demonstrated significantly less operation time compared to the periumbilical group, with a mean difference (MD) of −7.73 min (95% CI: −13.10 to −2.35). Importantly, the *I*^2^ value was reported as 0%, indicating no significant heterogeneity across these RCTs. In contrast, our study observed an average surgical time of 54 min for laparoscopic cholecystectomy using transumbilical access. While we did not directly compare this with alternative incision techniques, our observed surgical time falls within the range reported in the literature for laparoscopic cholecystectomy. However, it's worth noting that our study did not specifically investigate the comparison between transumbilical and periumbilical incisions. Therefore, direct comparison with the meta-analysis results may not be feasible.

Nonetheless, our findings provide valuable real-world data on the surgical time associated with transumbilical laparoscopic cholecystectomy for gallbladder diseases. This adds to the existing literature by offering insights into the practical application of transumbilical access in routine surgical practice.

According to our working group, the interpretation of this data cannot be disconnected from the surgeon's experience.

It is widely acknowledged that skilled laparoscopists can conduct surgeries more efficiently and accurately, resulting in fewer complications (24). However, while the learning curve for laparoscopic cholecystectomy remains undefined (25), the benefits of expertise in this field are well-established. In Lee et al. work (19), all surgeries were performed by a single team of highly experienced surgeons, while in our group, for each centre, the surgical team was composed by an expert surgeon and two residents. Besides, the gallbladder dissection is, in our centres, performed by leaving in place the outer layer of the gallbladder subserosal layer. The subserosal layer can, in fact, be divided into two sublayers, creating an avascular plane (25). This trick allows us to reduce the overall blood loss and the risk of vessel injuries; but on the other hand, it takes longer since it requires a higher precision.

Another substantial element to consider is that the umbilical scar is an anatomical and embryological point of interest, since in its correspondence the skin, the abdominal fascia and the parietal peritoneum are in direct contact, with not, or at least minimal, interposition of adipose tissue, even in obese subjects (15). This aspect implies that the TUL can be closed with a single layer suture (as shown in *Image 3*), while a periumbilical incision requires the opening of skin, subcutaneous adipose tissue and fascia, and each layer must be properly closed. It is our opinion that all these reasons are responsible for our longer, but still satisfying operative time (12). Moreover, in our patients, we also recorded the necessary time to accede the abdomen, with a mean result of 2.3 min. This data is, indeed, difficult to analyse since, despite the great number of attempts to establish the best entry technique, a minimal number of studies in literature reported this finding. In 2013 Angioli et al. compared three different entry techniques: Veress Needle, Direct trocar insertion and Open technique. They registered a mean entry time of respectively 212.4, 71.4 and 161.7 s. It is our opinion that further studies could be useful to evaluate this parameter (26).

Our study documented a low conversion rate to open surgery (0.55%, 14 cases) and minimal intraoperative complications, aligning with the findings reported by Lee et al. and Rafique et al. Shih et al.'s meta-analysis might have pooled data from studies utilizing various incision techniques, possibly obscuring the distinct outcomes associated with TUL.

Currently, the literature cites an overall conversion rate for laparoscopic cholecystectomy ranging from 1.84% to 4.90% (27, 28). The primary contributing factor to conversion to open surgery is often identified as a frozen Calot's triangle. However, a significant number of patients experience conversion to open surgery due to major vessel injury or bowel injury (29), which are common risks associated with the closed access technique using the Veress Needle (30). Both minor and major intraoperative complications resulting from Verres needle insertion have been documented over the years. Peterson et al. even documented a case of mortality attributed to abdominal aorta puncture (31). Additionally, vascular and visceral injuries are frequently documented, occurring at rates of 0.2 per 1,000 and 0.4 per 1,000, respectively (6, 32, 33). Furthermore, major vessel lesions have also been linked to Hasson trocar entry (33), albeit at lower rates compared to the closed entry technique (10). Certain studies comparing open and closed techniques for pneumoperitoneum induction even indicate that all entry-related fatalities occur in the Veress needle groups (33, 34).

In our subgroup of 14 patients, none required conversion to open surgery due to entry-related complications. The challenges leading to conversion in our cohort are outlined in Table 4. Notably, the incidence of major vessel injury during abdominal cavity access in our patient group was 0%. A recent review assessed the risk of major vessel injury using the Veress needle to be between 0.5% and 1.2%, with a significant mortality risk (10). The mean blood loss observed in our patients is 75 ml (range: 10–380 ml). This figure appears to be lower than the reported literature average, where blood loss is estimated at approximately 259.3 ± 188.5 ml (35). We attribute this outcome to TUL, as the absence of adipose tissue at the umbilical scar suggests a lack of blood vessels. However, a significant contribution to this outcome is also attributed to the gallbladder subserosal dissection method employed by our team.

Regarding the length of hospital stay, in the meta-analysis conducted by Shih et al., incorporating data from two randomized controlled trials (RCTs), the transumbilical group exhibited a non-significantly shorter duration compared to the periumbilical group, with a mean difference (MD) of −0.11 days (95% CI: −0.40 to 0.17). In contrast, our study documented an average hospital stay of 2.4 days for patients undergoing laparoscopic cholecystectomy via transumbilical access. While our findings suggest a relatively brief hospitalization period, it's important to note that our study did not directly compare the impact of different incision techniques on hospital stay duration. Therefore, direct comparison with the meta-analysis results may not be entirely appropriate.

Nevertheless, our results contribute to the understanding of postoperative recovery patterns following transumbilical laparoscopic cholecystectomy. The observed average hospital stay aligns with expectations for minimally invasive procedures and suggests a favorable postoperative recovery profile for patients undergoing laparoscopic cholecystectomy via transumbilical access. Further studies comparing hospital stay durations across various incision techniques could provide additional insights into the potential benefits of transumbilical access in terms of postoperative recovery and resource utilization.

The primary finding of this study is the remarkably low occurrence of port site hernias. Our research revealed an incidence rate of incisional hernias at 0.4%, with 10 out of 2,541 patients affected. Our findings demonstrate a lower incidence of incisional hernias compared to previous literature. In fact, the overall reported occurrence of port site hernias in the literature ranges from 0.3% to 5.4%, predominantly located at the previous periumbilical incision. Mayo et al. reported an incisional hernia rate of 1.6% in their study group (36), whereas Nassar et al. encountered trocar site hernias in 12% of their patients (37).

We hypothesized that the reduction in port site hernia rates among patients undergoing TUL is attributed to the unique anatomy and embryology of the umbilicus. The umbilical scar comprises only three layers: skin, aponeurotic fascia, and parietal peritoneum, without the presence of pre-peritoneal adipose tissue, even in obese individuals. Fathi et al., in a cadaver study, classified umbilical rings into five types based on the shape and attachment patterns of ligaments. They demonstrated an anatomical predisposition to umbilical hernia in patients lacking the umbilical fascia and with the round hepatic ligament unattached to the inferior border of the umbilical ring (38). Unfortunately, it is impossible to predict all these anatomical characteristics before surgery. We believe that TUL ensures a very low incidence of port site hernias also because the incision we perform is relatively short (approximately 15 ml) compared to a traditional periumbilical incision. The assessment of port site hernia risk percentages remains incomplete without considering another factor: wound infections. Presently, port site infection is widely acknowledged as one of the contributing factors in the pathogenesis of incisional hernias (39–41). Within our study group, 1.65% of patients (42 out of 2,543) experienced port site infections following TUL within the initial 10 postoperative days. All cases were conservatively managed with local Gentamicin treatment.

One of the primary concerns regarding TUL is the belief that the umbilical scar is more prone to bacterial colonization than other abdominal surfaces. However, prospective randomized trials comparing TUL and PUI demonstrate no significant difference in wound infection rates between the two groups (1, 41). Additionally, Hamzaoglu et al. demonstrated that povidone-iodine, routinely used before surgery, eradicated microorganisms in the umbilical dimple in 89% of patients. Furthermore, none of the microorganisms survived after povidone-iodine antisepsis, leading to wound infection in their patient cohort (42). In summary, within our study, all 10 patients who developed incisional hernias also experienced port site infections as a short-term postoperative complication, with the additional commonality of a high BMI among them.

None of the patients in our study group developed seroma as a short-term complication. This absence can be attributed to the direct contact between the aponeurotic fascia and the parietal peritoneum at the umbilical scar, where adipose tissue is absent (38, 43, 44).

Our results are consistent with the findings of the meta-analysis by Shih et al., which highlighted favorable outcomes with TUL. This convergence of findings across different studies underscores the robustness of evidence supporting the benefits of TUL in reducing postoperative complications. Furthermore, our study's outcomes are consistent with the trends observed in large RCTs, such as those by Bouffard-Cloutier et al. and Siribumrungwong et al., regarding reduced rates of incisional hernias with specific incision techniques.

One additional non-clinical observation we made pertains to cost savings. The incision made using the TUL technique measures approximately 15 ml in length, rendering the use of a Hasson trocar unnecessary; instead, we utilize a disposable 10- or 12-mm trocar. A single Hasson trocar costs around 115 Euros, inclusive of taxes. Conversely, a disposable 12 mm trocar costs approximately 75 Euros, taxes included—a savings of 35%. Across our cohort of 2,543 patients, this resulted in savings of approximately 101,720 Euros.

Our study benefits from a large sample size and detailed evaluation of TUL outcomes specifically in laparoscopic cholecystectomy for gallbladder diseases. By focusing exclusively on TUL, we provide targeted insights into the safety and efficacy of this technique in a specific surgical context. The consistency of our findings with those of prior studies, including large RCTs and meta-analyses, enhances the credibility of our results and their applicability to clinical practice.

However, our study differs from large randomized controlled trials (RCTs) and meta-analyses in several key aspects. While the meta-analysis examined various incision techniques across different laparoscopic procedures, our investigation specifically focuses on laparoscopic cholecystectomy for gallbladder diseases using the TUL approach exclusively. By concentrating on this specific indication, we aim to provide a comprehensive analysis of outcomes relevant to gallbladder surgery, offering more targeted and actionable insights for clinical practice. Furthermore, our study's emphasis on TUL in laparoscopic cholecystectomy allows for a detailed evaluation of its safety and efficacy in this context, particularly given the increasing interest in minimally invasive approaches for gallbladder surgery. Despite the strengths of our study, including its large sample size and comprehensive evaluation of TUL outcomes, certain limitations should be acknowledged. Specifically, the lack of a control group limits our ability to directly compare the efficacy of TUL with alternative access techniques. Additionally, the retrospective nature of our study introduces potential biases associated with data collection and analysis.

Nonetheless, notwithstanding these limitations, our study's strengths lie in its detailed analysis of TUL outcomes in laparoscopic cholecystectomy. By synthesizing evidence from a variety of sources, including large RCTs and meta-analyses, we provide valuable insights into the safety and efficacy of TUL in gallbladder surgery. This contributes to a better understanding of minimally invasive approaches and informs clinical decision-making in the management of gallbladder diseases. In conclusion, our study adds important insights to the existing literature on laparoscopic surgery for gallbladder diseases, particularly through its evaluation of the TUL approach. By leveraging evidence from prior studies, including those analyzed in the meta-analysis by Shih et al., we contribute to a more comprehensive understanding of the benefits and limitations of TUL in gallbladder surgery.

## Conclusions

In summary, our study underscores the safety, efficacy, and cost-saving potential of transumbilical laparoscopy (TUL) in laparoscopic cholecystectomy for gallbladder diseases. By providing targeted insights into this specific surgical approach, we contribute to the growing body of evidence supporting the adoption of TUL in clinical practice. Despite certain limitations, our findings add valuable depth to the literature, informing surgical decision-making and advancing patient care in the management of gallbladder diseases.

## Data Availability

The raw data supporting the conclusions of this article will be made available by the authors, without undue reservation.
